# Benefits and Meaning of Lipids Profile in Relation to Oxidative Balance and Brain Morphology in Schizophrenia

**DOI:** 10.3390/ijms241411375

**Published:** 2023-07-12

**Authors:** Natalia Śmierciak, Wirginia Krzyściak, Marta Szwajca, Paulina Karcz, Amira Bryll, Tadeusz J. Popiela, Paulina Donicz, Aleksander Turek, Veronika Aleksandrovych, Maciej Pilecki

**Affiliations:** 1 Department of Child and Adolescent Psychiatry, Faculty of Medicine, Jagiellonian University Medical College, 31-501 Krakow, Poland; natalia.smierciak@uj.edu.pl (N.Ś.); marta.szwajca@uj.edu.pl (M.S.); paulina.donicz@gmail.com (P.D.); maciej.pilecki@uj.edu.pl (M.P.); 2 Department of Medical Diagnostics, Jagiellonian University Medical College, 30-688 Krakow, Poland; wirginiakrzysciak@cm-uj.krakow.pl; 3 Department of Electroradiology, Jagiellonian University Medical College, 31-126 Krakow, Poland; paulina.karcz@uj.edu.pl; 4 Department of Radiology, Jagiellonian University Medical College, 31-501 Krakow, Poland; amira.bryll@uj.edu.pl (A.B.); msjpopie@cyf-kr.edu.pl (T.J.P.); 5 Doctoral School of Medical and Health Sciences, Jagiellonian University Medical College, 31-530 Krakow, Poland; alek.turek@doctoral.uj.edu.pl; 6 Department of Pathophysiology, Jagiellonian University Medical College, 31-121 Krakow, Poland

**Keywords:** schizophrenia, high-density cholesterol (HDL), lipids profile, brain morphology, oxidative stress, magnetic resonance

## Abstract

Schizophrenia is characterized by complex metabolic dysregulations and their consequences. Until now, numerous theories have explained its pathogenesis, using a spectrum of available technologies. We focused our interest on lipid profile—periphery high-density cholesterol level and lipoproteins in the human brain and compared magnetic resonance imaging (MRI) scans of patients with schizophrenia and the healthy group. Detailed analysis of biochemical parameters was performed using magnetic resonance spectroscopy. Our study aimed to reveal correlations between periphery high-density lipoproteins levels and lipoproteins in the brain, depicted in MRI scans, and parameters of peripheral oxidative stress expressed as paraoxonase. Patients with schizophrenia have decreased levels of high-density lipoproteins, low paraoxonase activity, and slightly raised sodium in the blood. Positive significant correlations between serum high-density cholesterol and anterior cingulate cortex, unique brain area for schizophrenia pathophysiology, MR spectroscopy signals, and diffusion have been revealed. To our knowledge, this is the first study to describe the effect of an anterior cingulate disorder on high-density cholesterol levels on the development of schizophrenia.

## 1. Introduction

Intracellular redox balance is crucial for neurophysiology and might be a trigger in the pathogenesis of mental diseases [1,2]. Undoubtedly, antioxidants are vital for maintaining brain homeostasis. Oxidative stress and its dysregulation play a significant role in the pathophysiology of schizophrenia (SZ) in endothelial dysfunction and atherosclerosis development [3]. Lower levels of paraoxonase 1 (PON-1) and peroxidase glutathione (GPx) are associated with abnormal expression of pro-inflammatory genes and their products, such as interleukins and cell adhesion molecules. Additionally, this decrease in antioxidant enzyme activity contributes to a reduction in anti-oxidative activity [4]. Intracellular adhesion molecule (ICAM), vascular cell adhesion molecules (VCAM), and monocyte chemoattractant protein-1 (MCP-1) promotes the infiltration of monocytes to the vascular wall and leads to an increase in local, causing endothelial dysfunction consequently—a key point of atherosclerotic plaque genesis [5,6].

There are still areas that are insufficiently explored and attract the attention of researchers. One such area pertains to the molecular mechanisms underlying the development of dyslipidemia in peripheral blood vessels and its impact on brain function and lipid levels within the central nervous system (CNS) during acute psychotic decompensation in individuals diagnosed with SZ. Based on the literature, the blood–brain barrier (BBB) is impermeable to lipids, and cholesterol is synthesized de novo in the CNS [7,8]. Furthermore, the brain contains about 20% of the whole body’s cholesterol and is the most cholesterol-rich organ [8]. Nevertheless, only high-density lipoproteins (HDL) have the capability to cross the blood–brain barrier (BBB) through a process called transcytosis [9]. This process involves HDL interacting with the scavenger receptor BI (SR-BI) present in the brain’s capillary endothelial cells [10]. SR-BI is known for its crucial role in HDL metabolism in the liver, but it is also present in the brain, facilitating the transport of HDL across the BBB. Low peripheral HDL levels may intensify inflammatory processes, increase the severity of atherosclerotic lesions in cerebral blood vessels [11,12] and influence the function of the endothelium in cerebral vessels, consequently affecting neuronal function. Cholesterol is crucial for various neuronal processes, including membrane integrity, synaptic function, and neurotransmitter receptor signaling. HDL helps regulate the distribution and transport of cholesterol within the CNS, ensuring a balance and proper availability of cholesterol for neuronal function [7]. In the context of SZ pathogenesis, multiple studies have explored the correlation between lipid profiles and the CNS physiology. Dyslipidemia, including alterations in HDL levels, has been associated with abnormalities in brain structure and function observed in SZ. Abnormal lipid metabolism and disturbances in cholesterol homeostasis may contribute to neurodevelopmental abnormalities, synaptic dysfunction, and impaired neurotransmission implicated in SZ.

Schizophrenia is a widespread chronic mental disorder, affecting about 1% of the world population [1], or up to 20 million people around the world [2]. Unfortunately, this complex disease with unknown etiology is still an actual medical and social problem in plenty of countries. Patients suffering from SZ no doubt have a poor prognosis, accompanied by a risk of somatic disorders and worsening psychiatric functioning. The primary identification of SZ is mostly based on common signs of psychopathological status, as well as behavioral and clinical symptoms [13,14]. The average age of SZ onset in men is 18–25, while in women it is between 25 and 35 years of age. The prodromal phase can be observed even up to 30 months before onset [13,15]. The life span of patients diagnosed with schizophrenia is 20 years shorter than the average of the general population [16]. During the last few decades, despite significant progress in medicine, the life span has not been elongated, but even become shorter. This observation is usually understood as a result of patients’ lifestyle, suicides, negative effects of pharmacotherapy, or limited access to treatment of somatic complications due to disease and socioeconomic status. The main risk factors in this regard are those associated with cardiometabolic complications. The most important in this context is the interaction between lipid metabolism disorders, oxidative balance disorders, and changes in peripheral vessels [2,17].

Schizophrenia is a complex mental disorder characterized by a wide range of symptoms. These symptoms are typically categorized into two groups: positive symptoms and negative symptoms. Positive symptoms are disturbances that are not typically present in healthy individuals (hallucinations, delirium, disorganized behavior), while negative symptoms involve the absence or reduction of normal behaviors and functions (anhedonia, a lack of movement, flat affect). The symptoms of SZ can be assessed using the Positive and Negative Syndrome Scale (PANSS) or the Brief Negative Symptoms Scale (BNSS) [2,18,19]. It is also unclear to what extent other mental disorders such as bipolar disorder, obsessive-compulsive disorder, or some of somatic disorders have a constellation of risk factors like SZ. For instance, the common feature in the pathogenesis of SZ and bipolar disorder might be immune activation. Subtle disorganization of thought processes and impairment of cognitive functions such as memory, attention, or learning may precede the appearance of a full-blown clinical syndrome by many months, if not years [1,13]. Uncertain etiology, a pallet of symptoms, and an inevitability of prognosis reflect difficulties of studying, emphasizing its necessity.

One of the important doubts appearing in the literature is to what extent schizophrenia is a disorder of the brain itself, manifested in the psychosocial functioning of the patient, and to what extent it is a systemic disorder in which pathophysiological phenomena concern not only the central nervous system but also the periphery. In recent years, the lipid profile, endothelial function, and role of oxidative stress have been widely discussed in the literature in the context of SZ pathogenesis as well as its spectrum disorders [12,18,20]. Metabolic disturbances are common in SZ. The lipid imbalance in such patients can be discussed in two different categories, sometimes combined: as side symptoms of treatment (some drugs lead to metabolic syndrome development) and as risk factor, impacted by severity and prognosis. Moreover, lipids may have their own role in inflammation development in SZ due to their involvement in oxidative stress in cells. The patients with first-episode psychosis without medication have lower levels of total cholesterol, low-density lipoproteins (LDL), and high-density lipoproteins (HDL), while their level of triglycerides is rising in comparison to healthy people. These changes were not related to age, gender, and body mass index (BMI) [21]. Patients with SZ have also altered antioxidant functions [22]. The interplay between lipid imbalance, oxidative stress, and inflammation may contribute to the increased vulnerability of individuals with SZ to cardiovascular complications.

Moreover, the first episode of SZ correlates with reductions in the essential omega-3 and omega-6 series of polyunsaturated fatty acids (PUFA) in erythrocytes membranes [20]. The same tendency was common for the pathogenesis of bipolar disorder [23]. The patients with first-episode psychosis demonstrated a declining in total oxidative resistance [24]. The role of oxidative stress in psychiatric diseases (anxiety, depression, schizophrenia, autism) has been observed, using animal models and deep analysis of brain metabolism [25,26].

Brain morphology and physiology make a neurobiological basis for genesis of psychiatric diseases. A comprehensive understanding of the intricate pathophysiological processes in brain disorders allows us to clearly identify key points in the genesis of schizophrenia. Thus, we have focused our attention on several main biochemical parameters reflects in brain physiology. The frontal cortex, temporal pole, dorsal anterior cingulate cortex (ACC), and putamen are involved in pathogenesis of SZ [27]. Lahutsina et al. suggested that the ACC gray matter reductions may presage psychosis onset [28]. Another study focused on the role of redox imbalance/mitochondrial dysfunction and implicate interneuron subtypes in ACC in SZ pathophysiology [29]. To note, patients with SZ have brains with a low pH (lower than normal) [30]. Probably, structural and biochemical changes in particular areas of the human brain may lead to oxidative imbalance and disturbances in dopaminergic and glutamatergic transmissions as consequences. The glutamatergic theory of psychosis has been described by our scientific group previously [31].

One of the most crucial parameters of lipid profile, which should be considered in analyses of risk factors and side effects in SZ, is HDL. Changes in HDL levels, whether declining or rising, may hold significance. As previously mentioned, decreased HDL levels have been observed in first-episode psychosis and SZ. Apolipoprotein A1 (ApoA1), as a component of HDL, and the antioxidative enzyme paraoxonase 1 (PON1), have anti-inflammatory, anti-atherogenic, antioxidant, and immunomodulatory roles. They are involved in the microenvironment in inflammations in the human body. Chronic SZ is characterized by an elevation of pro-inflammatory cytokines (interleukins (IL)-2, -6 and -10) [32,33,34,35].

Thus, inflammation may serve as a mechanism underlying the altered metabolism of lipids and, in conjunction with dyslipidemia, contribute to prefrontal cortex dysfunction, hippocampal impairments, and ultimately, the progression of psychotic symptoms in schizophrenia. We have hypothesized that dysfunction in the anterior cingulate gyrus (or in the prefrontal cortex) could be associated with lipid metabolism disorders during the first psychotic episode (FEP). However, evidence linking hippocampal alterations and lipid metabolism remains relatively limited. The aim of our innovative study was to comprehensively study the correlation between peripheral HDL levels and brain lipoproteins, as assessed by MRI scans, as well as peripheral oxidative stress as measured by paraoxonase.

## 2. Results

### 2.1. Clinical Characteristics of Schizophrenia Patients

The study was conducted on 40 people in the course of psychotic decompensation diagnosed with schizophrenia based on ICD-10 admitted to the In-Patient Unit at the Psychiatry Department. The period of untreated psychosis among patients (DUP) was a minimum of 3 weeks and a maximum of 140 weeks. The shortest hospital stay was 14 days; the longest was 171 days. The time of onset of FEP was at least 12 years and the most was 29 years, while the smallest number of psychotic episodes was 1, and the largest number was 15. The duration of the disease in years was, at the shortest, half a year, and the longest was 21 years. The clinical examination was based on the PANSS symptom scale (positive symptoms, negative symptoms, general functioning, total score scale). Among the 40 patients, the severity of positive symptoms ranged from a minimum of 25 points to a maximum of 70 points. The severity of negative symptoms ranges from a minimum of 15 points to a maximum of 38 points, and the level of general functioning ranges from a minimum of 12 points to a maximum of 39 points. The total scale ranges from a minimum of 69 to a maximum of 141. The detailed demographic and clinical characteristics of schizophrenia patients and were illustrated in Table 1. We assumed that DUP was the time of untreated psychosis as defined as the time from the onset of the first positive symptoms to the start of effective treatment in the course of the first psychotic episodes [36]. The current group of respondents is not the FEP (first psychotic symptoms) group, but a group that includes subsequent psychotic episodes, which does not allow for the assessment of DUP. DUP in this group of subjects was determined on the basis of the first medical documentation confirming FEP, where the time of untreated psychosis was recorded. Therefore, each patient, regardless of the number of episodes, on the basis of a medical examination from the first medical documentation documenting psychosis, has a certain time from the appearance of the first positive symptoms to the initiation of effective treatment.

### 2.2. Biochemical Blood Analysis

**Figure 1:**
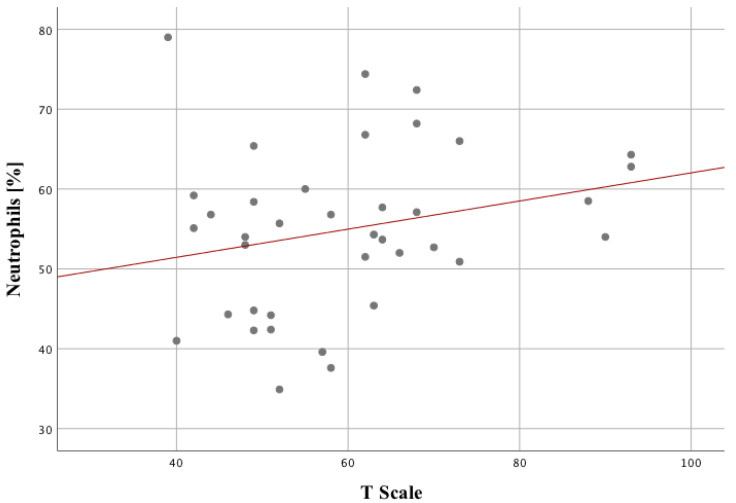
Relationship between the number of neutrophils and the T scale in the study group.

We have checked the statistically significant differences in sodium, HDL, and paroxonase-1 between the study and control groups. Post hoc test analysis showed that patients with SZ had decreased levels of HDL and PON-1 in comparison with the control group (Table 2). Effect size (eta-squared, eta-square eta^2^) demonstrated such parameters as: Sodium 0.23 (23%, Power = 99.7%), Glucose 0.12 (12%, Power = 86%), Cholesterol 0.08 (8%, power = 66.4%), HDL 0.09 (9%, power = 77.3%). Among the individual parameters, the factor that differentiates the two groups to the greatest extent is sodium. In the next step, it was checked the statistically significant relationships between blood count parameters and variables related to the quality of life in the studied group (Table 3). Among of many statistically significant correlations, the strong positive correlations of neutrophils were observed. Among the negative correlations, the strongest corelate was in the percentage of lymphocytes with G and N scores on admission to the hospital. The longer the duration of the disease, the higher the score on the T scale (Figure 1).

### 2.3. Correlation between PON-1 and Lipids in Both Observed Groups (the Control Group and Patients with Schizophrenia)

**Figure 2:**
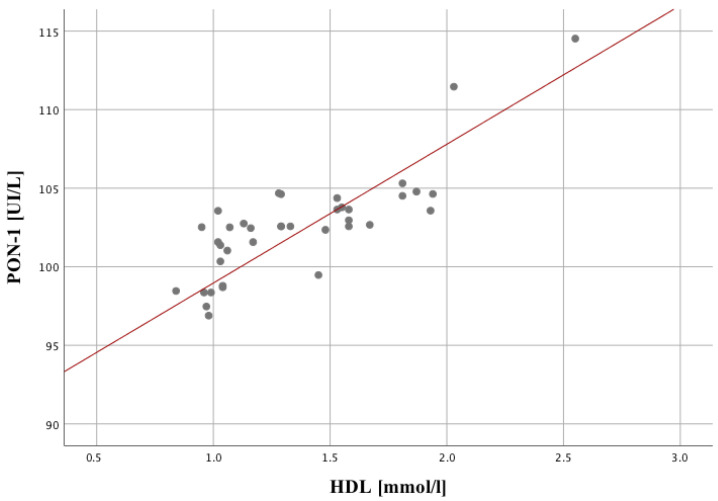
Correlation between LDL level and PON in the study group.

In the group of patients with psychosis in the course of schizophrenia, we observed a statistically significant positive correlation between PON-1 activity and serum HDL concentration (r = 0.82, *p* <0.01). Additionally, PON-1 activity correlated negatively with serum LDL concentration (r = −0.43, *p* < 0.01). Negative correlation has been observed for PON-1 and triglycerides (r = −0.44; *p* = 0.005). These tendencies have not been revealed in the control group. As for HDL, it shows several statistically significant relationships, the strongest of which concerns the first PON measurement (r = 0.81; *p* < 0.001) (Table 4). Interactions between PON and HDL were positive (the rising of one parameter correlates with an increasing a second one). LDL and HDL have also a strong correlation (r = −0.42; *p* = 0.007). The declining of the value of one variable is associated with increasing of another one (Figure 2).

**Figure 3:**
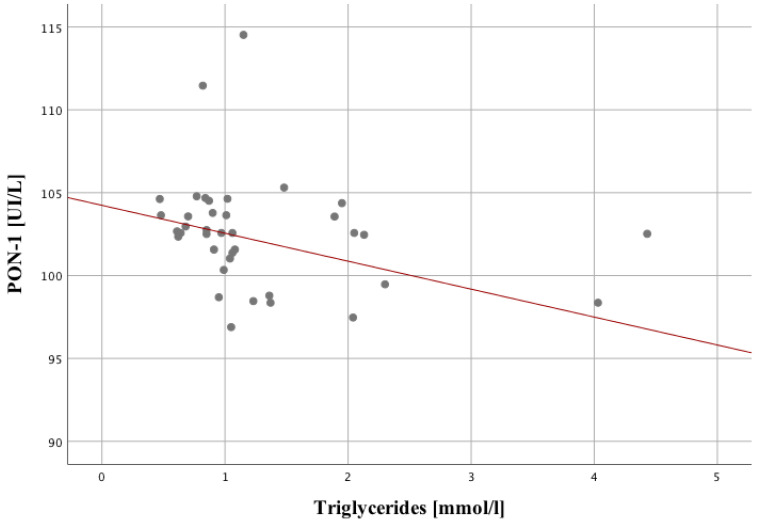
Correlation between triglycerides level and PON in the study group.

The level of triglycerides has the strongest correlation with the PON measured at hospital admission (r = −0.44; *p* = 0.005). The lower the value of one variable correlates with the higher for the other one (Figure 3).

### 2.4. MR Spectroscopy and Laboratory Measurements

In the group of patients, we observed positive significant correlations between serum HDL and anterior cingulate cortex MR spectroscopy signals: ACC-LIP (*p* < 0.01) and ACC-LIP/Cr (*p* < 0.05). In addition, a positive correlation was observed between HDL serum, GLC glucose level (*p* < 0.05), and myo-inositol Mi level in left frontal lobe (*p* = 0.004) and a negative correlation between HDL serum and CHO/CR ratio (*p* < 0.05). Alternatively, in the group of healthy controls, we observed significant positive correlations between serum HDL level and right frontal lobe MR spectroscopy signals: RFL-LIP (R = 0.39; *p* < 0.05) and RFL-LIP/Cr (R = 0.39; *p* < 0.05) (Table 5).

Analysis of the remaining lipid parameters in the study groups was performed, which obtained a negative relationship between the LDL serum and the ratio of alanine to creatine concentrations ALA/CR (*p* < 0.05), a negative relationship between the serum LDL and the ratio of glucose/creatine concentrations GLU/CR (*p* < 0.05), and positive between LDL serum and choline concentration (*p* < 0.05) in the F20 group (Table 6). In addition, in this group, a negative correlation was observed between serum triglycerides and the level of glutamate (GLU), glutamine (GLN) and gamma-aminobutyric acid (GSH) (R = −0.40, *p* < 0.05) and the level of glutamine (GLN) (R = −0.39 *p* < 0.05).

A negative relationship was found between serum HDL and diffusion (DWI) in the left cingulate cortex (ACC) (*p* < 0.05) and a positive relationship between serum HDL and diffusion (DWI) in the right anterior cingulate cortex MR (*p* < 0.05) (Table 7). The above tendencies were not observed in the control group.

The relationship between other lipid parameters and DWI was also analyzed in both groups. There was a positive relationship between diffusion in the right frontal lobe in the AVG sequence and a positive relationship between serum triglycerides and diffusion in the right anterior cingulate gyrus in the DEV sequence (Table 8).

**Figure 4:**
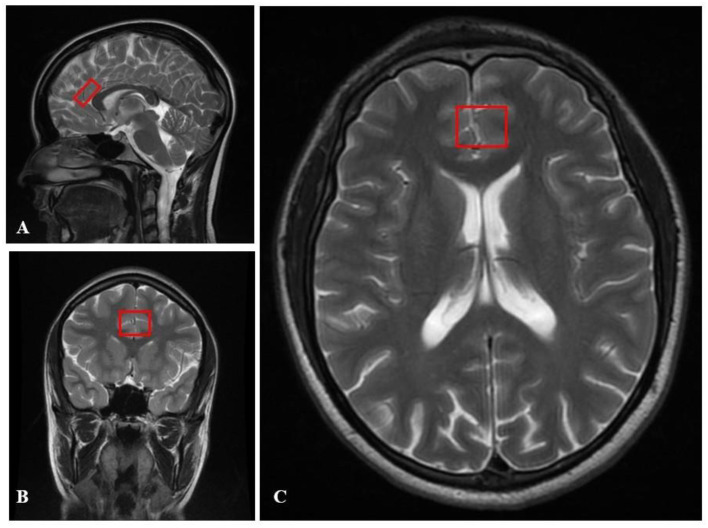
VOI location for spectroscopy in the human brain in MRI images. (**A**) the sagittal midline section; (**B**) the coronal section; (**C**) the transverse section.

**Figure 5:**
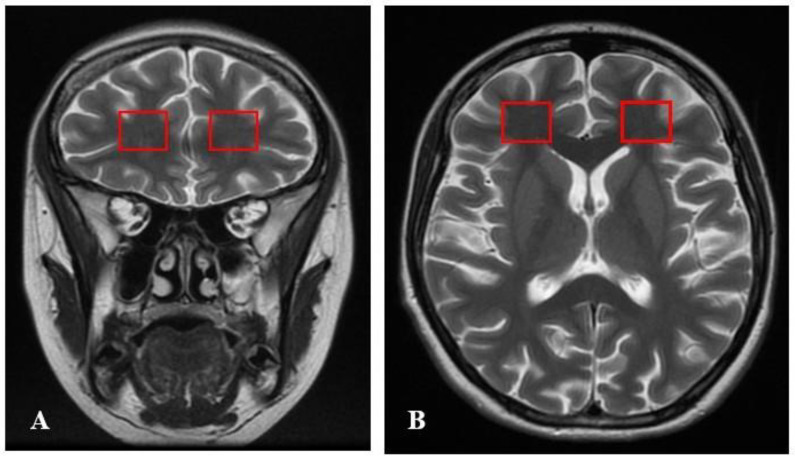
VOI location for spectroscopy in the human brain in MRI images. (**A**) the coronal section; (**B**) the transverse section.

**Figure 6:**
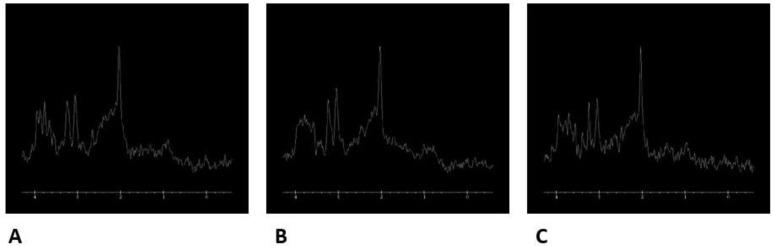
Spectroscopic spectrum in the patient from the study group: the right frontal lobe (**A**), the left frontal lobe (**B**) and the anterior cingulate cortex (ACC) (**C**).

**Figure 7:**
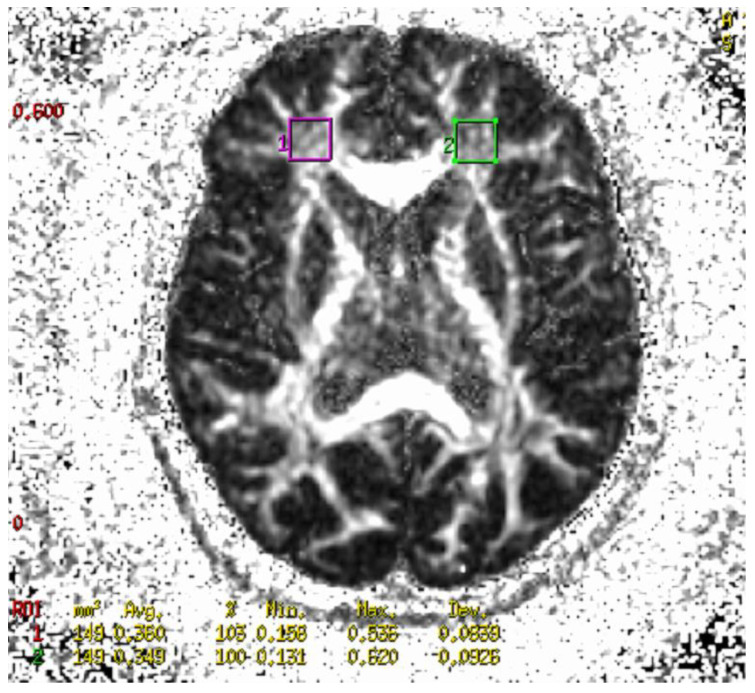
Fractional anisotropy map with marked ROI for measuring FA of patient with schizophrenia in the right and left frontal lobe.

**Figure 8:**
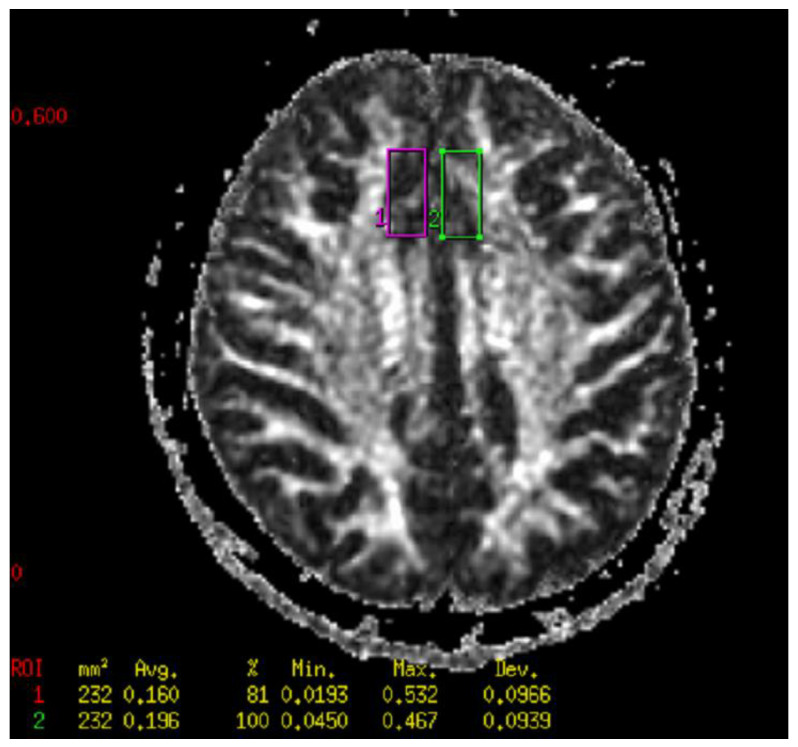
Fractional anisotropy map with marked ROI for measuring FA of patient with schizophrenia in the right and left anterior cingulate cortex.

Spectroscopic analysis has been performed in both observed groups in the frontal and ACC areas (Figure 4, Figure 5, Figure 6, Figure 7 and Figure 8). We want to present MRI scans and spectroscopic spectrum of the 17-year-old female patient. M.S. was urgently admitted to the In-Patient Unit at the Psychiatry Department of the University Hospital in Krakow due to bizarre behavior. According to her parents, the patient was agitated, experienced auditory and visual hallucinations, and voiced delusional content related to being followed by classmates who could read her mind. According to her parents, several months before the admission, she began to withdraw from peer contacts, stopped taking care of personal hygiene, and received worse grades at school. She periodically self-injured her forearms and was skipping school. A week before hospitalization, she was at a horse camp, where during one class she started uttering bizarre content, remained in great fear, and confirmed that she heard numerous male and female voices commenting on her person and urging her to commit suicide. She was brought by medical transport to the In-Patient Unit at the Psychiatry Department of the University Hospital in Krakow, where she was examined by a psychiatrist, and after excluding other reasons explaining the patient’s current condition, she was admitted to the In-Patient Unit at the Psychiatry Department of the University Hospital in Krakow with a preliminary diagnosis of acute psychosis. Before hospitalization, she did not take any medications; other diseases and the use of stimulants (drugs, cigarettes, alcohol, drugs) were excluded. The DUP time was calculated as 56 days. It was the first psychotic episode of the patient who was diagnosed with paranoid schizophrenia during hospitalization based on the ICD 10 diagnosis. On admission, the P scale—positive symptoms—received 34 points, the N scale—negative symptoms—28 points, the G scale—general symptoms—53, and the T scale—total scale—95. During the hospitalization, haloperidol was initially included in the treatment at a dose of 2 mg/day—in terms of Chlorpromazine (100 mg). The patient was included in the clinical trial. After 83 days, she was discharged from the Ward with a diagnosis of paranoid schizophrenia. At that time, she took olanzapine at a dose of 20 mg (in terms of chlorpromazine 400 mg) (Figure 4, Figure 5 and Figure 6).

## 3. Discussion

Psychiatric disorders are often accompanied by comorbidities (metabolic syndrome and cardiovascular diseases). Psychiatric patients may have an oxidative misbalance, which amplifies the risk of developing co-existing pathologies. The family of detoxifying enzymes, paraoxonases, includes three forms, and the first one (PON-1) is the most examined, while their role is still debatable. It is calcium-dependent enzyme, and is mostly expressed in the liver and in the kidneys [37,38], which are found in high-density lipoproteins (HDL) and in the intestinal cells [39]. PON-1 was studied in patients with anxiety disorders, bipolar disorders, major depressive disorders, obsessive compulsory disorders, a major depression, and schizophrenia. Our former studies revealed a positive correlation between PON-1 activity, HDL, and levels and the concentration of free triiodothyronine after drug treatment [37]. Based on the current literature data during the first episode of schizophrenia PON-1 activity is declined and is normalized only after appropriate therapy [38]. Maes et al. concluded that schizophrenia is associated with decreased levels of natural IgM and reduced PON-1 activity (antioxidative and anti-inflammatory) [40]. We have also observed lower activity of PON-1 in patients with SZ, which positively correlates with HDL levels and negatively correlates with LDL levels.

We suppose that oxidative imbalance and disturbances of immune microenvironment leads to neurodegeneration and manifestation of SZ, respectively. PON-1 enzymatic activity is associated with an intensity of kidney filtration: lower in patients with chronic kidney disease and hemodialysis [41]. In patients with hemodialysis elevation of CRP (C-reactive protein—the parameter of inflammatory process), it is correlated with PON-1 decline which reflects a risk of cardiovascular complications in these patients [42]. Our results demonstrate that the lowest level of PON-1 was in patients with schizophrenia, while the control group had higher concentrations. We can hypothesize that a decrease in PON-1 is associated with primary immunological disturbances and lower reparative and regenerative abilities. In such a group of people, the onset of schizophrenia could be observed. Based on data, PON-1 can transfer to the membrane, can be involved in HDL-mediated cholesterol outflow, and participate in the pathogenesis of oxidative stress-related diseases (including cardiovascular syndrome, HIV, metabolic syndrome) [39,43,44,45,46]. All known types of PON have different rations between gene expression and protein detecting, based on the type) of tissue and disease, but all of them modulate the inflammation and oxidative stress. PON-1 could be transported by HDL from blood circulation through the blood–brain barrier to the brain and the cerebrospinal fluid [39,47]. The reason for this transport is still not clear. However, brain-cholesterol homeostasis is crucial for neurodegeneration development. For instance, Alzheimer’s disease is associated with a damage of oligodendrocytes from one side, and compensatory intensified transport of PON-1 to oligodendrocytes to reduce the oxidative imbalance from another side [48]. The local inflammatory process leads to oxidative stress, in which PON-1 transported via HLD is involved as a force balancing redox reactions. Pro-inflammatory cytokines and neuroinflammation should be considered in the pathogenesis of SZ [36], in which patients have a thinner cerebral cortex compared to healthy people on average [48]. No doubt, mitochondrial dysfunction plays a role in the neurodegeneration in patients with neurological and psychiatric diseases (epilepsy, schizophrenia, multiple sclerosis, Alzheimer’s disease, Parkinson’s disease, Huntington’s disease).

The results obtained in this study are consistent with the results of the meta-analysis, which show that previously untreated patients with a first episode of non-affective psychosis showed significantly lower serum total cholesterol, LDL, and HDL levels compared to healthy controls [49]. Lipid levels are very important for membrane balance, which is essential for myelin integrity [50] and synaptic transmission [7]. Although peripheral blood cholesterol cannot cross the blood–brain barrier (BBB), evidence suggests that there is a leakage between central and peripheral lipid metabolism that may ultimately affect central cholesterol concentrations [7,51]. The possible mechanism of the altered lipid profile in schizophrenia may be due to overlapping genetic risk factors for schizophrenia and the LDL and HDL profile [52]. Gjerde et al. stressed that increasing HDL level during antipsychotic therapy is associated with improvement in negative symptoms in first-episode psychosis [53,54,55,56]. Another study depicted that abnormal HDL level might be an early-onset event in drug-naïve first-episode psychosis of adolescents and young adults who are unlikely to have other cardiometabolic risks [32]. The relation between negative syndromes of SZ and metabolic abnormalities was observed by Wójciak et al.: women have a significant positive correlation between negative symptoms and HDL level, while men do not [33].

Confounding factors such as drugs and disease duration may be involved, which together pose a problem in identifying altered connectivity between peripheral and central lipid metabolism. Our findings may reveal pathological changes in schizophrenia. In addition, structural analysis of prefrontal cortex regions including subfields of the hippocampus revealed reduced volume in first-episode psychotic patients. This result is consistent with other reports that emphasize that smaller hippocampal volume is already evident in the early stages of the first psychotic episode [57].

In the presented study, one of the most interesting findings is the significant association between changes in total cholesterol and HDL levels and functional connectivity in the prefrontal cortex, which is in close proximity to the hippocampus. This association explains the lower cholesterol levels observed in schizophrenia patients, which are linked to higher prefrontal-hippocampal and thalamic-hippocampal connectivity. Membrane lipids, including cholesterol, are believed to be essential in maintaining membrane stability and neuronal function. Previous studies have shown that the synthesis and catabolism of cholesterol in the brain proceed dynamically in neurons affected by glutamate-mediated excitotoxicity, especially in the hippocampus [58]. These findings closely correspond to our own results, which indicate that changes in glutamatergic transmission in the anterior cingulate gyrus turned out to be crucial in differentiating patients with schizophrenia [31].

Furthermore, the observed negative correlation between serum HDL and DWI in the left cingulate cortex may indicate a potential mechanism, which can be explained by the fact that the lowered HDL cholesterol level is associated with increased functional connectivity in the prefrontal cortex in patients with schizophrenia. To the best of our knowledge, this study is the first to investigate the association between anterior cingulate disorder and high-density cholesterol levels in individuals with schizophrenia. Both in vitro and in vivo studies have shown that long-term exposure to excitatory neurotransmission can cause loss of membrane cholesterol in prefrontal cortex neurons [58]. For instance, the study found a loss of up to 34% of cholesterol after 7 days of monosodium L-glutamate injection in the hippocampus of adult rats [59]. The results indicate that treatment with high doses of NMDA agonists can then result in the lowering of cholesterol in hippocampal synaptosomes and loss of membrane cholesterol, accompanied by the upregulation and translocation of cholesterol-24-hydroxylase (Cyp46A1) to the surface of neurons; consequently, the site of the transition of cholesterol I to 24S-hydroxycholesterol and removes cholesterol from neurons. In such a situation, accelerated cholesterol loss may result in degeneration of the neural membrane, myelin, and axons of sensitive areas of the brain, indicating the disrupted integrity of the thalamic-hippocampal junctions also seen in our study. Astrocytes and microglia, both types of glial cells are essential for the nervous system, and insufficiency leads to neurodegeneration. Pathophysiology of SZ includes abnormalities in glial cells, especially astrocytes [60]. Notter concluded that they can impact neurodevelopmental and homeostatic processes, connected with SZ pathogenesis [61].

Other environmental factors, such as poor eating habits characterized by the consumption of high-calorie foods or saturated fatty acids, have been implicated in the development of neural imbalances and may contribute to the risk of schizophrenia. It is hypothesized that these dietary factors can disrupt the delicate balance of cholesterol in the brain, leading to dysfunction in critical brain regions involved in schizophrenia pathology. Specifically, the association between lowered serum cholesterol levels and dysfunction in the thalamic-hippocampal junction suggests a dynamic interplay between neuronal excitotoxicity responses and altered cholesterol balance during the early stages of schizophrenia.

According to our hypothesis, dysfunction in the anterior cingulate gyrus (or in the prefrontal cortex) is associated with lipid metabolism disorders during the psychotic episode. Among all metabolic markers, we observed significant intergroup differences in serum total and HDL cholesterol levels. There are few studies indicating the occurrence of subclinical dyslipidemia associated with low total cholesterol levels observed in patients with a first psychotic episode. This supports the hypothesis that lower cholesterol levels may be phenotypically important for people with previously untreated psychotic disorders. Similar observations were made in previously untreated patients prior to psychosis who had lower levels of total cholesterol, cholesterol esters, and phospholipids in the plasma membranes of skin fibroblasts [62].

Our results are consistent with a meta-analysis in which previously untreated patients with a first episode of psychosis had significantly lower serum total cholesterol and HDL compared to healthy controls [49]. Lipid levels are crucial for maintaining the balance of lipid membranes, which are an integral part of myelin [50]. The possible mechanism of the altered lipid profile in schizophrenia may be due to the overlapping risk factors for schizophrenia, the broad spectrum of disease, and redox levels including HDL and PON-1 [63].

**Figure 9:**
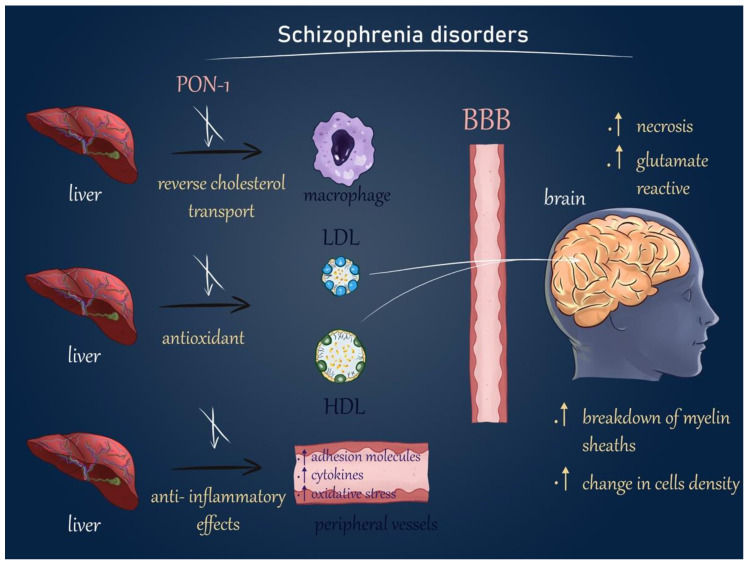
Central and peripheral actions of HDL in patients with schizophrenia disorders. PON-1 activity is decreased in patients in a psychotic decompensation comparison with healthy people. We hypothesized that PON-1 may be a dysfunctional unit, which has not protected HDL or LDL from oxygenation and thus loses anti-inflammatory effects. Hence, there has been a rise in the intensity of oxidative stress manifestations, adhesion molecules, and cytokines concentrations in the peripheral blood vessels. It has also enhanced cholesterol accumulation in macrophages. Dysfunctional and oxidized HDL may be transported in the human brain (in the anterior cingulate gyrus), where it can contribute to local necrotic reactions, stimulate glutamate reactivity, and impact the breakdown of myelin sheaths and cell density. In summary, the dysregulation of HDL and PON-1 in patients with schizophrenia may result in dysfunctional HDL with impaired protective effects and increased oxidative stress. This can have implications for both peripheral and central processes, potentially contributing to neuroinflammation, myelin disruption, and altered cell density in specific brain regions such as the anterior cingulate gyrus. PON-1—paraxonase 1; HDL—high density lipids; LDL—low density lipids; BBB—blood brain barrier.

Patients with SZ exhibit distinct lipid profiles characterized by decreased activity of paraoxonase-1, an enzyme essential for HDL metabolism. We hypothesized that SZ is accompanied by dysfunctional HDL existence and physiological disturbances in some areas of brain, passively involved in SZ pathogenesis (Figure 9).

Spectrum of lipids in ACC reflects tiny focuses of necrosis or local myelin damage, leading to immunological misbalance and decreasing anti-oxidative activity consequently. It might be also considered as a risk factor of cardiovascular diseases in patients with SZ. We hope that this current study will help reveal a linkage between lipid balance and pathogenesis of psychiatric disorders.

## 4. Materials and Methods

### 4.1. Participants of the Study

The study was conducted on patients with schizophrenia (F20 according to version 10 of the International Statistical Classification of Diseases) in the course of psychotic decompensation admitted to the In-Patient Unit for Adults and In-Patient Unit for Adolescents at the Psychiatry Department of the University Hospital in Krakow. The study group was comprised of 22 men, 18 women; age range 15–38; mean age 22.68 ± 7.39. Patients were recruited from January 2019 to January 2020. The study was conducted in accordance with the moral, ethical, regulatory, and scientific principles governing clinical research. The study was conducted according to the guidelines of the Declaration of Helsinki and approved by the Bioethics Committee of the Jagiellonian University protocol 1072.6120.152.2019 of 27 June 2019, 1072.6120.17.2020 of 27 February 2020 and 1072.6120.252.2021 of 17 January 2022. Patient data and results of diagnostic tests were stored and processed in an anonymous form. Each patient participating in the study gave informed consent to participate in the planned research and received a protocol with a detailed description and plan of the study. At any stage of the study, the participant was allowed to withdraw from the research study. None of the respondents took advantage of this possibility. The control group has been presented by 30 volunteers aged between 18 and 40 (17 women and 13 men; average age 29.5 ± 5.33 years) without any psychiatric diseases. The characteristics of these groups were described previously by Śmierciak et al. [37].

### 4.2. The Assessment of the Clinical Status and Pharmacotherapy

Each participant of the study obtained demographic data, a detailed medical history, perinatal and early childhood history, data about hereditary diseases, the presence of accompanying somatic diseases, and previously used pharmacotherapy or illegal drugs. Symptoms of psychosis in the group of people with psychotic decompensation were assessed using the PANSS diagnostic scale of positive and negative symptoms [38]. This scale is consisting of four subscales: positive symptoms scale (P); negative symptoms scale (N); general psychopathology scale (G); and scale describing the total result (T). In the differential diagnosis group, the clinical state was assessed by a physician and a psychologist based on the presented symptoms in accordance with ICD-10.

### 4.3. Pharmacotherapy

Of note, 32 patients received monotherapy, and 8 patients received two-drug polytherapy. The most commonly used drug in monotherapy was haloperidol (18 patients). It was also used in 5 cases of polytherapy. The second most frequently used monotherapy drug was olanzapine. Quetiapine (2 patients), aripiprazole (2 patients), and risperidone (2 patients) were also used as monotherapy. In four cases of polytherapy, olanzapine was added to haloperidol and in one case to aripiprazole. In single patients there were combinations of quetiapine with aripiprazole, olanzapine with quetiapine, and olanzapine with aripiprazole. Drug doses were converted to chlorpromazine equivalents. Chlorpromazine equivalent (CPZE) is defined as a drug dose that corresponds to 100 mg of an oral dose of chlorpromazine [43]. A drug equivalent to 200–300 mg of chlorpromazine is considered a minimally effective dose, while more than 1000 mg of chlorpromazine is considered high. None of the patients experienced side effects.

### 4.4. Metabolic Profiles

Peripheral blood samples were collected by clot-activating gel-containing serum separator tubes at the laboratory of the University Hospital in Krakow Poland and processed before 8 a.m. on the same day. All participants fasted for 8–12 h before a blood test. Routine analyses were performed using XN-2000 automated analyzers (Sysmex Corp., Kobe, Japan) as well as Cobas 6000 and Cobas 8000 biochemical analyzers (Roche HoldinG, Basel, Switzerland)/(Roche Diagnostics, Mannheim, Germany). Routine blood laboratory tests included blood morphology, lipid profile: triglycerides (TG), total cholesterol, high-density lipoproteins (HDL), low-density lipoproteins (LDL), C-reactive protein (CRP), ionogram (K^+^, Na^+^, Mg^2+^), and glucose.

### 4.5. Measurement of the Activity of Paraoxonase 1 (PON-1) in the Blood

The enzymatic activity of paroxonase was assessed by method of Eckerson et al. with own modification described in our previous paper [35,43].

### 4.6. Neuroimaging Analysis

The study used magnetic resonance imaging (MRI) with a 1.5 T magnetic field induction MR system and an 8-channel head coil in a horizontal position to obtain images of the brain. A standard MR brain examination protocol was used, which included sequences for FLAIR, T1-weighted, T2-weighted, and diffusion-weighted imaging (DWI). Assessment of brain morphology was performed in order to exclude pathological or congenital lesions. Additional analysis of diffusion in the ACC region was performed using the Functool (version 7.0) image analysis software (the version 7.0) (GE Healthcare; Chicago, IL, USA) [31]. During this study, the standard protocol for MRI of the head in force at the University Hospital in Krakow was used. This is already written in version 495–496. The PRESS sequence was used to select the region of interest in spectroscopy (described in version 502–507). The MR system was equipped with firm whole-body gradients that provided an amplitude of 33 mT/m and a rising rate of 120 T/m/s on each axis, which allowed for highly repeatable, fast, and accurate scans. Two-dimensional T2-weighted images were acquired in sagittal, axial, and coronal planes to show the position of the volume of interest (VOI) and anatomical brain structures for spectroscopy.

Magnetic resonance spectroscopy (MRS) was performed using the single-voxel technique (SVS) with a point-resolved spectroscopy sequence (PRESS) and chemical shift selective imaging sequence (CHESS) for water suppression. Automatic shimming was used to achieve satisfying spectra, which is an automatic procedure to improve the homogeneity of the magnetic field after a patient is placed in it and applies to the volume of the selected voxel (voxel shimming). During the project, MRS signal was obtained from three locations situated symmetrically in the anterior cingulate cortex (ACC), right and left frontal lobes, centered on the interhemispheric fissure and parallel and superior to the dorsal anterior surface of the corpus callosum. The VOI reached approximately 8 cm^3^ and was adapted to the anatomical size of the location where the spectrum was obtained from. The period of the sequence lasted 2 min 12 s, and MRS acquisition specifications of 35 ms TE with 64 averages were obtained.

In the study described, MRS was used to investigate the metabolic changes in the anterior cingulate cortex (ACC) and frontal lobes of the brain. The data obtained from MRS were processed using SAGE 7.0 (Spectroscopy Analysis by GE). The processing involved reconstruction of the data, offset correction, zero filling, apodization, determination of peaks, Fourier transformation, and calculation of relative ratios of metabolite concentrations in relation to Cr.

The processing steps involved converting the Free Induction Decay (FID) signal into numerical data files containing basic (raw) data. The raw data were then reconstructed by applying filters and automatic Fourier transformation. The reconstructed data were subjected to offset correction, zero filling, and apodization to improve the signal-to-noise ratio and resolution of the spectrum. Peaks in the spectrum were identified and marked for further analysis.

The spectrum with marked peaks was then converted back into the FID signal, which was Fourier transformed to obtain the final spectrum. The area under the selected peaks was then read, and the developed spectrum was superimposed on the original spectrum to confirm the accuracy of the analysis. Finally, the relative ratios of metabolite concentrations in relation to Cr were calculated.

Metabolites were manually chosen from the spectrum: lipids (lip 0.9–1.0 ppm), lactates (lac 1.33 ppm), alanine (ala 1.8), N-acetyl-aspartate (NAA 2.02 ppm), glutamate (glu 2.1 ppm), γ-aminobutyric acid (GABA 2.3 ppm), glutamine (gln 2.45 ppm), creatine (Cr 3.02 ppm), choline (Cho 3.22 ppm), glucose (glc 3.43 and 3.8 ppm), myoinositol (mI 3.56 and 4.0 ppm), glu + gln + GSH complex (3.7 ppm), and phosphocreatine (3.9 ppm).

Diffusion imaging (DWI) was performed using EPI sequences. The EPI sequence uses two strong magnetic field gradients, called diffusion gradients. Diffusion gradients are symmetrically switched on before and after the pulse 180° in a single spin-echo sequence. The task of diffusion gradients is to distinguish between diffusing and stationary spins. The first diffusion gradient causes the spins to phase apart, the second to re-phase. If the spins cannot diffuse freely, they are affected by both diffusion gradients. As a result, the MR signal is strengthened. In the case of free diffusion, the phasing is not complete, which causes attenuation of the MR signal amplitude. The DWI sequence consisted of two acquisitions for two different values of the b factor. One acquisition was made for b = 0, the other for b = 1500 (b is a technical parameter of diffusion gradients). DWI sequence parameters are presented in Table 9. The process of diffusion in tissues should be extended to include phenomena occurring in biostructures, such as perfusion, microcirculation, movement on a macroscopic scale (respiratory movements, heartbeat, patient movement), and movement at the tissue level (mainly related to capillary blood flow as well as tissue pulsation in the vicinity of larger arteries). All these phenomena, together with diffusion anisotropy in the brain tissue, affect the amplitude of the MR signal. Therefore, instead of the diffusion coefficient used in physics and chemistry, the term ADC is used. In addition, the parameter affecting the contrast of the DWI image is the echo time. Typically, TE = 60–120 ms is used to visualize the diffusion process in the tissue. This means that the signal in the DWI images also depends on the transverse relaxation time of the tissue. In order to obtain images that depend only on the diffusion process, the value of the apparent diffusion coefficient (ADC) was calculated for each voxel of the imaged layer. The values of the ADC coefficient for individual voxels are presented using the so-called ADC maps. The ADC map consists of pixels whose color depends on the value of the ADC coefficient. The higher the ADC value, the brighter the pixel on the ADC map, and vice versa. The value of the ADC coefficient can be determined numerically. For this purpose, additional software installed on the diagnostic station is used. The degree of orientation of the movement of water molecules, i.e., the so-called fractionated anisotropy, was examined using diffusion tensor imaging (DTI). Sequence parameters are presented in Table 9. In order to express the degree of anisotropy of the examined structure, the fractional anisotropy (FA) coefficient was calculated. It is a scalar quantity reaching values in the range of 0–1. A value of 0 corresponds to an isotropic structure, and a value of 1 to a structure in which diffusion is only possible in one direction. Due to the degree of organization, the white matter of the brain is characterized by a high value of FA (the highest in the corpus callosum and pyramidal tracts). Gray matter is characterized by relatively low FA values. Very low (close to 0) FA values are typical for cerebrospinal fluid. MR imaging enables the measurement of the diffusion process of water molecules in human tissues, particularly the brain. The diffusion of water molecules in the white matter and spinal cord is anisotropic, while it is isotropic in the gray matter. The source of anisotropy is due to the cell membranes and the degree of myelination of fibers. With an increase in the degree of myelination (maturation) of nerve cells, there is an increase in diffusion anisotropy and a decrease in the average diffusion coefficient. These changes can be explained by an increase in myelination, a reduction in the amount of water in the brain, an increase in the cohesion and compactness of nerve fibers, and a reduction in intercellular space observed with age. In addition, damage or degeneration of nerve fibers leads to a decrease in the degree of anisotropy. Diffusing molecules move between tissue microstructures, reflecting or passing through various barriers in the form of cell membranes or cell organelles. These barriers impede the free diffusion of water molecules. The displacement of water molecules in the tissue depends on the microstructure of the tissue. A short diffusion time reflects the local viscosity of the intracellular space, while a longer time reflects the cellular barrier effect. Therefore, the measurement of the DWI signal and the ADC value reflects the tissue microstructure, and the magnitude of the ADC depends on the type of tissue. The source DWI and DTI images were quantified using FuncTool 7.0 software for the reconstruction and analysis of DICOM images on the GE Advantage Workstation 4.5 diagnostic station. The analysis of the source DWI and DTI data was carried out in the following stages: (1) entering the source DWI and DTI images into FuncTool, (2) setting the &quot; Processing Threshold&quot; to the maximum value to ensure that all pixels within the brain were selected for the calculation of the final ADC maps, (3) checking the value of the parameter b read from the DICOM file, (4) automatic generation of ADC and FA maps, and (5) measuring the DWI signal, ADC, and FA coefficients in particular places. The DWI, ADC, and FA values in the FuncTool software are displayed as mean values with standard deviation. The DWI, ADC, and FA values were obtained from three locations situated symmetrically in the anterior cingulate cortex (ACC), right and left frontal lobes, centered on the interhemispheric fissure and parallel and superior to the dorsal anterior surface of the corpus callosum (Figure 7 and Figure 8).

### 4.7. Statistical Analysis

Statistical analysis was performed using the IBM SPSS Statistics 25.0 package. Kruskal–Wallis test analysis allowed for the checking of whether there are statistically significant differences between more than two groups of people. When statistically significant differences occurred, an appropriate post hoc test was used. This allowed for the checking of which groups there are statistically significant differences. The selection was made on the basis of homogeneity of variance in the compared groups. In the case of comparing two groups, it was the Mann–Whitney U test. Spearman’s correlation analysis made it possible to check whether there is a statistically significant relationship between the variables studied. Regression analysis allowed to build a statistically significant model that has the greatest impact on the quality of life of the surveyed people. The value of *p* < 0.05 was considered statistically significant.

### 4.8. Limitations

The present study has several limitations that should be acknowledged. Firstly, the small group size is a result of the exploratory nature of the study. Consequently, the findings may not be generalizable to a larger population. Furthermore, the participants in the study are at different stages of illness and have experienced varying numbers of psychotic episodes. Additionally, there is a range of clinical symptom severity within the group of subjects, while the control group comprises subjects who are comparatively older. It is worth noting that due to the limited group size, it was not possible to conduct an analysis stratified by gender, which could be an important variable to consider.

Another significant limitation is the absence of analyses regarding the impact of pharmacotherapy on the pathophysiological processes being investigated. The use of neuroleptics in the study group introduces heterogeneity, as these medications have varying effects on lipid metabolism and the cardiovascular system. Moreover, potential factors that could influence oxidation and oxidative processes, such as developmental factors (e.g., birth complications), were not taken into account. The researchers also did not consider other potential confounding factors, including diet and lifestyle. Additionally, changes in body weight, BMI, and waist circumference, as well as the assessment of cardiovascular parameters, were not included in the study, which limits a comprehensive understanding of the potential relationships between the variables under investigation.

## 5. Conclusions

To summarize, our study reveals significant findings regarding the lipid profile and oxidative balance in patients with schizophrenia. We found that individuals with schizophrenia exhibit alterations in lipoprotein levels, particularly decreased HDL and PON-1 activity. These findings suggest the presence of dysfunctional HDL and compromised anti-oxidative activity in this patient population. The correlation between serum HDL concentration and PON-1 activity, as well as the negative correlation with LDL concentration, further the involvement of lipoproteins in the pathophysiology of schizophrenia.

Furthermore, our research highlights the significance of brain imaging, particularly MRI scans and diffusion abnormalities in the anterior cingulate gyrus or prefrontal cortex, in establishing a link between the biochemical profile and the risk of schizophrenia. The observed disruptions in diffusion and localized disturbances in lipid spectra may indicate the presence of necrotic foci or myelin damage, contributing to immune dysregulation and reduced antioxidant activity within specific brain regions. In our suggestions, we are also based on data, presented by the scientific groups [64,65,66,67].

Overall, our findings suggest that lipid abnormalities and oxidative imbalance may serve as potential risk factors for both the onset of schizophrenia and the development of cardiovascular diseases in affected individuals. Further investigations are warranted to unravel the underlying mechanisms and explore potential therapeutic strategies targeting lipid metabolism and oxidative stress in the context of schizophrenia.

## Figures and Tables

**Table 1:** Descriptive statistics of variables characteristic of the disease history for the studied group. Duration of untreated psychosis (DUP) and medical history data concerned the group of schizophrenia patients; DUP was defined as the time from the onset of psychotic symptoms until the first treatment. Assessment of the clinical state based on the PANSS (Positive and Negative Syndrome Scale). Abbreviations: M—mean; Me—median; SD—standard deviation; SD—standard deviation; DUP—Duration of untreated psychosis; PANSS P—PANSS psychotic symptoms subscale; PANSS N—PANSS Negative symptoms subscale; PANSS G—PANSS Global symptoms subscale.

	Minimum	Maximum	Mean	SD
DUP (weeks)	3	140	24.3	27.59
Hospitalization (days)	14	171	64.23	33.16
Age of first episode	12	29	18.78	3.75
Number of episodes	1	15	3.45	3.79
Duration of illness (years)	0.5	21	3.84	5.66
PANSS P	25	70	54.12	10.56
PANSS N	15	38	27.87	6.68
PANSS G	12	39	25.70	5.53
PANSS Total	69	141	107.70	20.23

**Table 2:** The difference in biochemical parameters in two observed groups of patients (effect size–eta-square, eta^2^).

Variable	M		SD		Me		Result of a Statistical Test
	Control Group	SZ	Control Group	SZ	Control Group	SZ	
Sodium [mmoL/L]	138.73	140.05	1.74	1.93	139	140	g2(2) = 23.34; *p* < 0.001
HDL [mmoL/L]	1.62	1.35	0.43	0.4	1.63	1.29	g2(2) = 9.52; *p* = 0.002
PON-1 [UI/L]	117.5	102.54	2.21	1.73	117.4	101.8	g2(2) = 76.4; *p* = 0.03

**Table 3:** Linkage between biochemical parameters and clinical condition assessed using the PANSS scale.

Variable	Scale P	Scale N	Scale T	Scale G
Neut [×10^3^/µL]	0.4; *p* = 0.01	0.4; *p* = 0.01	0.4; *p* = 0.01	0.49; *p* < 0.001
Neut [%]	0.46; *p* <0.001	0.47; *p* < 0.001	0.48; *p* < 0.00	0.55; *p* < 0.001
Lymph [%]	−0.4; *p* = 0.01	−0.45; *p* < 0.001	−0.46; *p* < 0.001	−0.51; *p* < 0.001
CRP [mg/L]	0.32; *p* = 0.04	0.19	0.31	0.3
Potassium [mM/L]	−0.04	−0.9	−0.03	−0.36; *p* = 0.03
Cholesterol [mM/L]	0.08	0.35; *p* = 0.03	−0.07	0.09

**Table 4:** The relation between lipids parameters and concentration and PON-1.

	HDL	LDL	Cholesterol	Triglycerides
PON-1	816 **	−0.424 **	−0.134	−0.442 **

**—*p* ≤ 0.01.

**Table 5:** The relation between HDL parameters and brain parameters in the study group.

Variable	ACC/LIP 0.9–1.0	ACC/Lip/Cr	GLC	Mi 3.56	CHO/CR
HDL	0.477 **	0.406 *	0.396 *	0.5	−0.360 *

ACC-LIP—concentration of lipids—fragment of necrosis in the area of the anterior cingulate cortex (ACC); ACC/Lip/Cr—lipid/creatine concentration in the area of the anterior cingulate cortex (ACC); GLC—glucose concentration; Mi—myo-inositol; CHO/CR—choline to creatine ratio. *—*p* ≤ 0.05; **—*p* ≤ 0.01.

**Table 6:** The relation between other lipids parameters and brain parameters in the study group.

	ALA/CR	GLU/CR	CHO 3.22	GLU + GLN + GSH 3.7	GLN 2.45
LDL [mmoL/L]	−0.376 *	−0.420 *	0.367 *	−0.235	−0.068
Cholesterol [mmoL/L]	0.01	− 0.14	−0.195	0.198	0.029
Triglycerides [mmoL/L]	0.066	−0.154	−0.164	−0.404 *	−0.389 *

Ala/Cr—alanine to creatine ratio; Glu/Cr—glucose to creatine ratio; CHO—cholinę concentrtion; Glu + GLN + GSH—concentration of glutamate, glutamine of gamma aminobutyric acid; GLN—glutamine concentration. *—*p* ≤ 0.05.

**Table 7:** The relation between HDL and the brain diffusion in the study group.

	FA_ACC_LEFT_AVG	ADC_ACC_RIGHT_DEV
HDL	−0.402 *	0.346 *

*—*p* ≤ 0.05.

**Table 8:** Correlation between another lipid’s parameters and brain diffusion.

	DWI_FRONTAL AREA_LEFT_AVG	DWI_FRONTAL AREA_RIGHT_AVG	ADC_ACC_RIGHT_DEV
LDL [mmoL/L]	0.373 *	0.387 *	0.051
Triglycerides [mmoL/L]	0.196	0.111	0.346 *
Cholesterol [mmoL/L]	0.138	0.213	0.12

*—*p* ≤ 0.05.

**Table 9:** Diffusion imaging and diffusion tensor imaging sequence parameters.

Parameter	Value	
	DWI	DTI
Scan plane	AXIAL	AXIAL
Sequence	EPI	EPI
Number of slice	22–24	22–24
Repetition time TR (ms)	8400	8300
Echo time TE (ms)	106	110
Field of view (cm)	24	20
Slice of thickness (mm)	5.0	5.0
Spacing (mm)	1.5	1.5
Matrix	128 × 128	128 × 128
Number of acquisition	2	2
Max parameter value $b\;\left(s/{mm}^{2}\right)$	1500	1500
Min parameter value $b\;\left(s/{mm}^{2}\right)$	0	0
Number of dynamic measurement directions	3	25

## Data Availability

Not applicable.

## References

[1] Freedman R. (2003). Schizophrenia. N. Engl. J. Med..

[2] Buosi P., Borghi F.A., Lopes A.M., Facincani I., Fernandes-Ferreira R., Oliveira-Brancati C., do Carmo T.S., Souza D., da Silva D., de Almeida E.A. (2021). Oxidative stress biomarkers in treatment-responsive and treatment-resistant schizophrenia patients. Trends Psychiatry Psychother..

[3] Zhang X.Y., Zhou D.F., Cao L.Y., Zhang P.Y., Wu G.Y., Shen Y.C. (2004). Changes in serum interleukin-2, -6, and -8 levels before and during treatment with risperidone and haloperidol: Relationship to outcome in schizophrenia. J. Clin. Psychiatry.

[4] Lucero D., Islam P., Freeman L.A., Jin X., Pryor M., Tang J., Kruth H.S., Remaley A.T. (2020). Interleukin 10 promotes macrophage uptake of HDL and LDL by stimulating fluid-phase endocytosis. Biochim. Biophys. Acta Mol. Cell Biol. Lipids.

[5] Ermakov E.A., Dmitrieva E.M., Parshukova D.A., Kazantseva D.V., Vasilieva A.R., Smirnova L.P. (2021). Oxidative Stress-Related Mechanisms in Schizophrenia Pathogenesis and New Treatment Perspectives. Oxidative Med. Cell. Longev..

[6] Ogłodek E.A. (2017). The role of PON-1, GR, IL-18, and OxLDL in depression with and without posttraumatic stress disorder. Pharmacol. Rep. PR.

[7] Zhang J., Liu Q. (2015). Cholesterol metabolism and homeostasis in the brain. Protein Cell.

[8] Björkhem I., Meaney S., Fogelman A.M. (2004). Brain cholesterol: Long secret life behind a barrier. Arterioscler. Thromb. Vasc. Biol..

[9] Fung K.Y., Wang C., Nyegaard S., Heit B., Fairn G.D., Lee W.L. (2017). SR-BI Mediated Transcytosis of HDL in Brain Microvascular Endothelial Cells Is Independent of Caveolin, Clathrin, and PDZK1. Front. Physiol..

[10] Tosheska Trajkovska K., Topuzovska S. (2017). High-density lipoprotein metabolism and reverse cholesterol transport: Strategies for raising HDL cholesterol. Anatol. J. Cardiol..

[11] Iqbal F., Baker W.S., Khan M.I., Thukuntla S., McKinney K.H., Abate N., Tuvdendorj D. (2017). Current and future therapies for addressing the effects of inflammation on HDL cholesterol metabolism. Br. J. Pharmacol..

[12] Hottman D.A., Chernick D., Cheng S., Wang Z., Li L. (2014). HDL and cognition in neurodegenerative disorders. Neurobiol. Dis..

[13] Jauhar S., Johnstone M., McKenna P.J. (2022). Schizophrenia. Lancet.

[14] Ochoa S., Usall J., Cobo J., Labad X., Kulkarni J. (2012). Gender differences in schizophrenia and first-episode psychosis: A comprehensive literature review. Schizophr. Res. Treat..

[15] Kaliuzhna M., Kirschner M., Carruzzo F., Hartmann-Riemer M.N., Bischof M., Seifritz E., Tobler P.N., Kaiser S. (2020). Clinical, behavioural and neural validation of the PANSS amotivation factor. Schizophr. Res..

[16] Wildgust H.J., Hodgson R., Beary M. (2010). The paradox of premature mortality in schizophrenia: New research questions. J. Psychopharmacol..

[17] Koman-Wierdak E., Róg J., Brzozowska A., Toro M.D., Bonfiglio V., Załuska-Ogryzek K., Karakuła-Juchnowicz H., Rejdak R., Nowomiejska K. (2021). Analysis of the Peripapillary and Macular Regions Using OCT Angiography in Patients with Schizophrenia and Bipolar Disorder. J. Clin. Med..

[18] Murray A.J., Rogers J.C., Katshu M., Liddle P.F., Upthegrove R. (2021). Oxidative Stress and the Pathophysiology and Symptom Profile of Schizophrenia Spectrum Disorders. Front. Psychiatry.

[19] Kott A., Daniel D. (2018). T56. An Exploratory Analysis Converting Scores between the Panss and Bnss. Schizophr. Bull..

[20] De-Oliveira J.L., da-Silva I.R., Ramis T.R., Ferreira C.V., Soares S.M., Ribeiro J.L., Dorneles G.P., Wagner L.C. (2018). Endothelial function and lipid profile of individuals with schizophrenia participating in a supported employment program. Rev. Bras. Med. Trab..

[21] Pruett B.S., Meador-Woodruff J.H. (2020). Evidence for altered energy metabolism, increased lactate, and decreased pH in schizophrenia brain: A focused review and meta-analysis of human postmortem and magnetic resonance spectroscopy studies. Schizophr. Res..

[22] Rambaud V., Marzo A., Chaumette B. (2022). Oxidative Stress and Emergence of Psychosis. Antioxidants.

[23] Pillinger T., Beck K., Stubbs B., Howes O.D. (2017). Cholesterol and triglyceride levels in first-episode psychosis: Systematic review and meta-analysis. Br. J. Psychiatry J. Ment. Sci..

[24] McNamara R.K., Welge J.A. (2016). Meta-analysis of erythrocyte polyunsaturated fatty acid biostatus in bipolar disorder. Bipolar Disord..

[25] Perry B.I., McIntosh G., Weich S., Singh S., Rees K. (2016). The association between first-episode psychosis and abnormal glycaemic control: Systematic review and meta-analysis. Lancet Psychiatry.

[26] Kriisa K., Haring L., Vasar E., Koido K., Janno S., Vasar V., Zilmer K., Zilmer M. (2016). Antipsychotic Treatment Reduces Indices of Oxidative Stress in First-Episode Psychosis Patients. Oxidative Med. Cell. Longev..

[27] Sukumar N., Sabesan P., Anazodo U., Palaniyappan L. (2020). Neurovascular Uncoupling in Schizophrenia: A Bimodal Meta-Analysis of Brain Perfusion and Glucose Metabolism. Front. Psychiatry.

[28] Lahutsina A., Spaniel F., Mrzilkova J., Morozova A., Brabec M., Musil V., Zach P. (2022). Morphology of Anterior Cingulate Cortex and Its Relation to Schizophrenia. J. Clin. Med..

[29] Kondo M.A., Norris A.L., Yang K., Cheshire M., Newkirk I., Chen X., Ishizuka K., Jaffe A.E., Sawa A., Pevsner J. (2022). Dysfunction of mitochondria and GABAergic interneurons in the anterior cingulate cortex of individuals with schizophrenia. Neurosci. Res..

[30] Park H.J., Choi I., Leem K.H. (2021). Decreased Brain pH and Pathophysiology in Schizophrenia. Int. J. Mol. Sci..

[31] Bryll A., Krzyściak W., Karcz P., Pilecki M., Śmierciak N., Szwajca M., Skalniak A., Popiela T.J. (2021). Determinants of Schizophrenia Endophenotypes Based on Neuroimaging and Biochemical Parameters. Biomedicines.

[32] Zhai D., Lang Y., Feng Y., Liu Y., Dong G., Wang X., Cao Y., Cui T., Ni C., Ji Y. (2017). Early onset of cardiometabolic risk factor profiles in drug naïve adolescents and young adults with first-episode schizophrenia. Schizophr. Res..

[33] Wójciak P., Domowicz K., Rybakowski J.K. (2021). Metabolic indices in schizophrenia: Association of negative symptoms with higher HDL cholesterol in female patients. World J. Biol. Psychiatry Off. J. World Fed. Soc. Biol. Psychiatry.

[34] Balõtšev R., Koido K., Vasar V., Janno S., Kriisa K., Mahlapuu R., Ljubajev U., Parksepp M., Veiksaar P., Volke V. (2017). Inflammatory, cardio-metabolic and diabetic profiling of chronic schizophrenia. Eur. Psychiatry J. Assoc. Eur. Psychiatr..

[35] Na K.S., Jung H.Y., Kim Y.K. (2014). The role of pro-inflammatory cytokines in the neuroinflammation and neurogenesis of schizophrenia. Prog. Neuro-Psychopharmacol. Biol. Psychiatry.

[36] Cechnicki A., Hanuszkiewicz I., Polczyk R., Bielańska A. (2010). Prospektywna ocena wpływu czasu nie leczonej psychozy na przebieg schizofrenii. Psychiatr. Pol..

[37] Śmierciak N., Szwajca M., Popiela T.J., Bryll A., Karcz P., Donicz P., Turek A., Krzyściak W., Pilecki M. (2022). Redefining the Cut-Off Ranges for TSH Based on the Clinical Picture, Results of Neuroimaging and Laboratory Tests in Unsupervised Cluster Analysis as Individualized Diagnosis of Early Schizophrenia. J. Pers. Med..

[38] Moreira E.G., Boll K.M., Correia D.G., Soares J.F., Rigobello C., Maes M. (2019). Why Should Psychiatrists and Neuroscientists Worry about Paraoxonase 1?. Curr. Neuropharmacol..

[39] Salazar J.G., Marsillach J., Reverte I., Mackness B., Mackness M., Joven J., Camps J., Colomina M.T. (2021). Paraoxonase-1 and -3 Protein Expression in the Brain of the Tg2576 Mouse Model of Alzheimer’s Disease. Antioxidants.

[40] Maes M., Vojdani A., Geffard M., Moreira E.G., Barbosa D.S., Michelin A.P., Semeão L.O., Sirivichayakul S., Kanchanatawan B. (2019). Schizophrenia phenomenology comprises a bifactorial general severity and a single-group factor, which are differently associated with neurotoxic immune and immune-regulatory pathways. Biomol. Concepts.

[41] Connelly P.W., Yan A.T., Nash M.M., Wald R.M., Lok C., Gunaratnam L., Kirpalani A., Prasad G. (2021). The Increase in Paraoxonase 1 Is Associated With Decrease in Left Ventricular Volume in Kidney Transplant Recipients. Front. Cardiovasc. Med..

[42] Kannampuzha J., Darling P.B., Maguire G.F., Donnelly S., McFarlane P.C.T., Chan C.T., Connely P.W. (2010). Paraoxonase 1 arylesterase activity and mass are reduced and inversely related to C-reactive protein in patients on either standard or home nocturnal hemodialysis. Clin. Nephrol..

[43] Taylor D.M., Barnes T.R.E., Young A.H. (2021). The Maudsley Prescribing Guidelines in Psychiatry.

[44] Eckerson H.W., Romson J., Wyte C., La Du B.N. (1983). The human serum paraoxonase polymorphism: Identification of phenotypes by their response to salts. Am. J. Hum. Genet..

[45] Moniczewski A., Gawlik M., Smaga I., Niedzielska E., Krzek J., Przegaliński E., Pera J., Filip M. (2015). Oxidative stress as an etiological factor and a potential treatment target of psychiatric disorders. Part 2. Depression, anxiety, schizophrenia and autism. Pharmacol. Rep..

[46] Prabakaran S., Swatton J.E., Ryan M.M., Huffaker S.J., Huang J.T.-J., Griffin J.L., Wayland M., Freeman T., Dudbridge F., Lilley K.S. (2004). Mitochondrial dysfunction in schizophrenia: Evidence for compromised brain metabolism and oxidative stress. Mol. Psychiatry.

[47] Wills A.-M., Landers J.E., Zhang H., Richter R.J., Caraganis A.J., Cudkowicz M.E., Furlong C.E., Brown R.H. (2008). Paraoxonase 1 (PON1) Organophosphate Hydrolysis Is Not Reduced in ALS. Neurology.

[48] Li C., Yang T., Ou R., Shang H. (2021). Overlapping Genetic Architecture Between Schizophrenia and Neurodegenerative Disorders. Front. Cell Dev. Biol..

[49] Misiak B., Stańczykiewicz B., Łaczmański Ł., Frydecka D. (2017). Lipid profile disturbances in antipsychotic-naive patients with first-episode non-affective psychosis: A systematic review and meta-analysis. Schizophr. Res..

[50] Mighdoll M.I., Tao R., Kleinman J.E., Hyde T.M. (2015). Myelin, myelin-related disorders, and psychosis. Schizophr. Res..

[51] Vitali C., Wellington C.L., Calabresi L. (2014). HDL and cholesterol handling in the brain. Cardiovasc. Res..

[52] Holven K.B., Retterstøl K., Ueland T., Ulven S.M., Nenseter M.S., Sandvik M., Narverud I., Berge K.E., Ose L., Aukrust P. (2013). Subjects with low plasma HDL cholesterol levels are characterized by an inflammatory and oxidative phenotype. PLoS ONE.

[53] Gjerde P.B., Dieset I., Simonsen C., Hoseth E.Z., Iversen T., Lagerberg T.V., Lyngstad S.H., Mørch R.H., Skrede S., Andreassen O.A. (2018). Increase in serum HDL level is associated with less negative symptoms after one year of antipsychotic treatment in first-episode psychosis. Schizophr. Res..

[54] Morris G., Puri B.K., Bortolasci C.C., Carvalho A., Berk M., Walder K., Moreira E.G., Maes M. (2021). The role of high-density lipoprotein cholesterol, apolipoprotein A and paraoxonase-1 in the pathophysiology of neuroprogressive disorders. Neurosci. Biobehav. Rev..

[55] Smith J., Griffiths L.A., Band M., Horne D. (2020). Cardiometabolic Risk in First Episode Psychosis Patients. Front. Endocrinol..

[56] Marthoenis M., Martina M., Alfiandi R., Dahniar D., Asnurianti R., Sari H., Nassimbwa J., Arafat S.M.Y. (2022). Investigating Body Mass Index and Body Composition in Patients with Schizophrenia: A Case-Control Study. Schizophr. Res. Treat..

[57] Seidman L.J., Kremen W.S., Koren D., Faraone S.V., Goldstein J.M., Tsuang M.T. (2002). A comparative profile analysis of neuropsychological functioning in patients with schizophrenia and bipolar psychoses. Schizophr. Res..

[58] Sodero A.O., Vriens J., Ghosh D., Stegner D., Brachet A., Pallotto M., Sassoè-Pognetto M., Brouwers J.F., Helms J.B., Nieswandt B. (2012). Cholesterol loss during glutamate-mediated excitotoxicity. EMBO J..

[59] Zhang X., Xu N., Li J., Ma Z., Wei L., Liu Q., Liu J. (2020). Engineering of L-glutamate oxidase as the whole-cell biocatalyst for the improvement of α-ketoglutarate production. Enzym. Microb. Technol..

[60] Wu Y., Chen M., Jiang J. (2019). Mitochondrial dysfunction in neurodegenerative diseases and drug targets via apoptotic signaling. Mitochondrion.

[61] Notter T. (2021). Astrocytes in schizophrenia. Brain Neurosci. Adv..

[62] Mahadik S.P., Mukherjee S., Correnti E.E., Kelkar H.S., Wakade C.G., Costa R.M., Scheffer R. (1994). Plasma membrane phospholipid and cholesterol distribution of skin fibroblasts from drug-naive patients at the onset of psychosis. Schizophr. Res..

[63] Andreasen N.C., Liu D., Ziebell S., Vora A., Ho B.C. (2013). Relapse duration, treatment intensity, and brain tissue loss in schizophrenia: A prospective longitudinal MRI study. Am. J. Psychiatry.

[64] Rachel W., Siwek M., Dudek D., Zięba A., Werewka-Maczuga A., Herman-Sucharska I., Urbanik A. (2008). Magnetic resonance proton spectroscopy in affective disorders. Neuropsychiatr. Neuropsychol./Neuropsychiatry Neuropsychol..

[65] Pathmasiri K.C., Pergande M.R., Tobias F., Rebiai R., Rosenhouse-Dantsker A., Bongarzone E.R., Cologna S.M. (2020). Mass spectrometry imaging and LC/MS reveal decreased cerebellar phosphoinositides in Niemann-Pick type C1-null mice. J. Lipid Res..

[66] Sibtain N.A., Howe F.A., Saunders D.E. (2007). The clinical value of proton magnetic resonance spectroscopy in adult brain tumours. Clin. Radiol..

[67] Kato T., Inubushi T., Kato N. (1998). Magnetic resonance spectroscopy in affective disorders. J. Neuropsychiatry Clin. Neurosci..

